# Supplementary material for: Rapid 3D phenotypic analysis of neurons and organoids using data-driven cell segmentation-free machine learning
Source: PLoS Comput Biol. 2021 Feb 22;17(2):e1008630. doi: 10.1371/journal.pcbi.1008630 (PMC7932518; doi:10.1371/journal.pcbi.1008630)
Supplement: S1 Text — Fig A. Small molecule inhibition of Bcl-2 family proteins in neurons. A) Sample images of primary cortical neurons treated with the indicated compounds illustrate the difficulty in observational assessment of drug responses in dense cultures. Prior to imaging the cells were stained with mitochondrial outer membrane potential-sensitive dye Mitotracker (red), the apoptosis indicator annexin V labeled with FITC (green) and the nuclear stain Hoechst (blue). To assess the cell death responses of cells visually it is important to be able to relate nuclear condensation to changes in mitochondrial transmembrane potential or annexin V staining for the same cell, which is not possible in images of neurons at this density. Alternative measures such as Western blotting are not amenable to high-throughput or the small number of cells in one well of a 384 well plate. Images are for visual inspection with channels scaled to equal intensities and were not used for analysis. Scale bar: 100 μm. B, C) Variation in phenotypic response of primary cortical neurons to different compound treatments from three plate replicates. Two dimensional principal component projection of the Phindr3D feature space of 3D multichannel images for the common treatments from the three different plates. Each point represents one 3D multichannel image. Here, we only analyzed conditions that were common across all plate replicates. These data allow visual confirmation of the minimal variability across three plate replicates, encompassing a total of nine biological replicates (i.e. cortical neurons derived from 9 separate animals). We further computed average values of Phindr3D features for each combination of compound treatment and concentration in each plate and computed Euclidean distances between the average profiles resulting in a distance vector for a plate. A high correlation implies treatments are distributed similarly for each plate. Next, we calculated the correlation of distance vectors between each [file pcbi.1008630.s001.pdf]

<u>S1 Text:</u> Supporting Information

Rapid 3D phenotypic analysis of neurons and organoids using data-driven cell segmentation-free machine learning

Philipp Mergenthaler[1,2,3¶*], Santosh Hariharan[1,4¶], James M. Pemberton[1,4], Corey Lourenco[4,5], Linda Z. Penn[4,5], David W. Andrews[1,4*]

[1]Biological Sciences, Sunnybrook Research Institute, University of Toronto, Toronto, Ontario, Canada.
[2]Charité – Universitätsmedizin Berlin, Dept. of Experimental Neurology, Dept. of Neurology, Center for Stroke Research Berlin, NeuroCure Clinical Research Center, Berlin, Germany.
[3]Berlin Institute of Health (BIH), Berlin, Germany.
[4]Department of Medical Biophysics, University of Toronto, Toronto, Ontario, Canada
[5]Princess Margaret Cancer Centre, University Health Network, University of Toronto, Toronto, Ontario, Canada

*Corresponding authors
Email: philipp.mergenthaler@charite.de (PM), david.andrews@sri.utoronto.ca (DWA)

¶These authors contributed equally to this work.

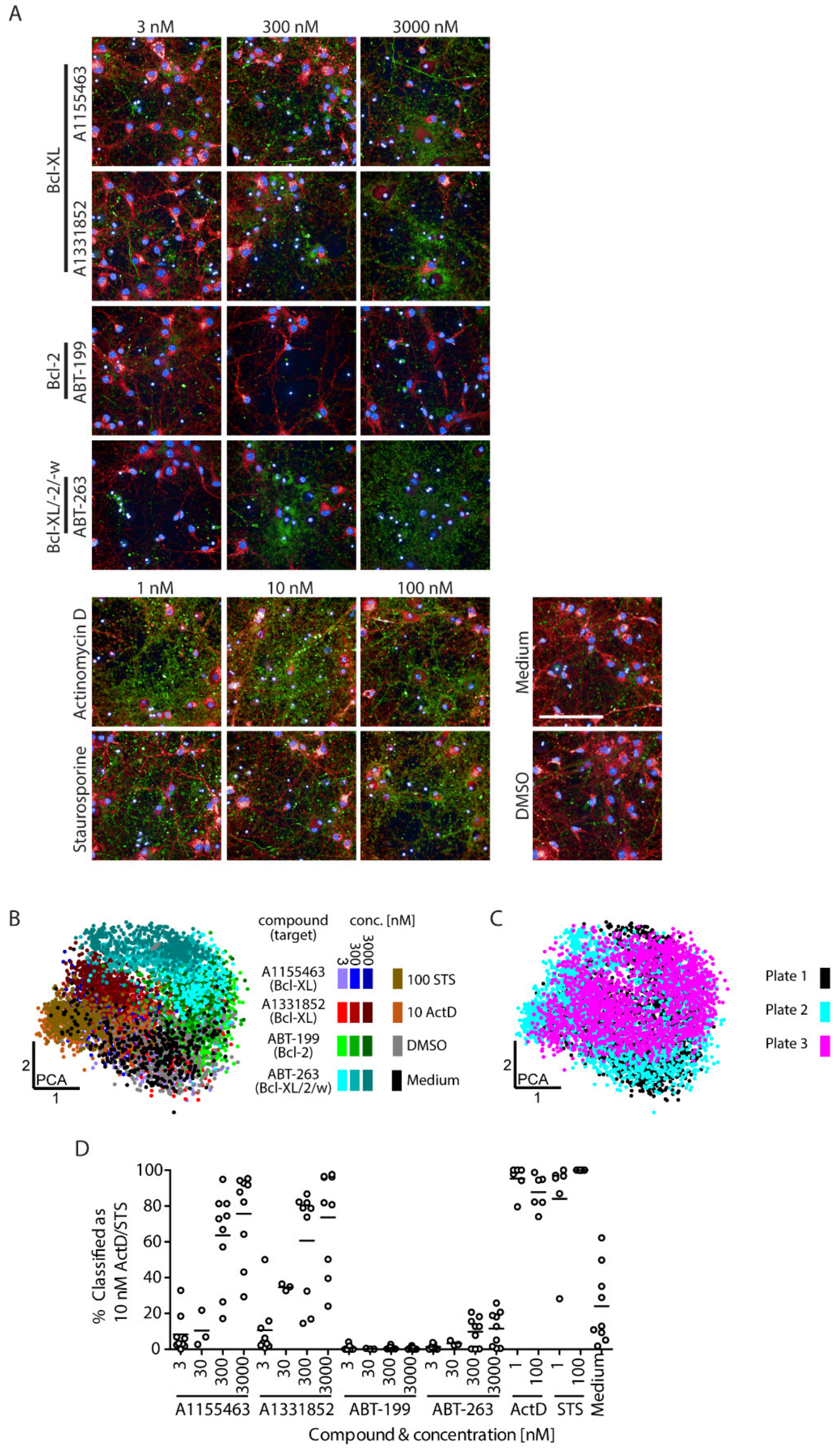

Fig A: Small molecule inhibition of Bcl-2 family proteins in neurons.

A) Sample images of primary cortical neurons treated with the indicated compounds illustrate the difficulty in observational assessment of drug responses in dense cultures. Prior to imaging the

cells were stained with mitochondrial outer membrane potential-sensitive dye Mitotracker (red), the apoptosis indicator annexin V labeled with FITC (green) and the nuclear stain Hoechst (blue). To assess the cell death responses of cells visually it is important to be able to relate nuclear condensation to changes in mitochondrial transmembrane potential or annexin V staining for the same cell, which is not possible in images of neurons at this density. Alternative measures such as Western blotting are not amenable to high-throughput or the small number of cells in one well of a 384 well plate. Images are for visual inspection with channels scaled to equal intensities and were not used for analysis. *Scale bar:* 100 μm.

B, C) Variation in phenotypic response of primary cortical neurons to different compound treatments from three plate replicates. Two dimensional principal component projection of the Phindr3D feature space of 3D multichannel images for the common treatments from the three different plates. Each point represents one 3D multichannel image. Here, we only analyzed conditions that were common across all plate replicates. These data allow visual confirmation of the minimal variability across three plate replicates, encompassing a total of nine biological replicates (i.e. cortical neurons derived from 9 separate animals). We further computed average values of Phindr3D features for each combination of compound treatment and concentration in each plate and computed Euclidean distances between the average profiles resulting in a distance vector for a plate. A high correlation implies treatments are distributed similarly for each plate. Next, we calculated the correlation of distance vectors between each plate. This resulted in correlation coefficient values of 0.74 (Plate1-Plate2), 0.85 (Plate 1 – Plate 3) and 0.88 (Plate 2-Plate 3), confirming minimal variability across the plate replicates. **B)** The colors correspond to the different treatment groups indicated on the right. **C)** The colors correspond to replicate plates as indicated at the right.

D) Classification based concentration response accuracy depends on training sets. Concentration response of primary cortical neurons treated with different anti-apoptotic protein inhibitory compounds calculated based on classification with a random forests classifier trained on DMSO negative controls and 10 nM actinomycin D (ActD) and 10 nM staurosporine (STS) positive controls (10 nM ActD/STS). Experimental means are indicated with horizontal lines, dots represent individual 3D image stacks. Phindr3D features were extracted from pooled images from three replicate plates. Images were then classified as either DMSO (alive) or ActD/STS (dead).

While increasing concentrations of A1155463 and A1331852 resulted in a corresponding increase in the number of images classified as ActD/STS, this was the case for increasing concentrations of ABT-263 only to a minor extent. The results for ABT-263 are most similar to those for untreated neurons (Medium). However, Phindr3D correctly identified inhibition of Bcl-2 by ABT-199 as a separate phenotype. Finally, the Phindr3D analysis trained on DMSO detected that some of the untreated cells (Medium) were not identical to the DMSO class illustrating the inherent heterogeneity within the cultures detected also by clustering (Figs 3 and 4) and the sensitivity of the method as well as the unpredictable classification of data that do not match a training class precisely. The discrepancy of this result compared to the high degree of cell death seen in the images (panel A) of the high concentrations of ABT-263 suggests that while visually recognizable as dead, the morphologies of these cells are not similar to cells treated with ActD/STS as further discussed in the main text.

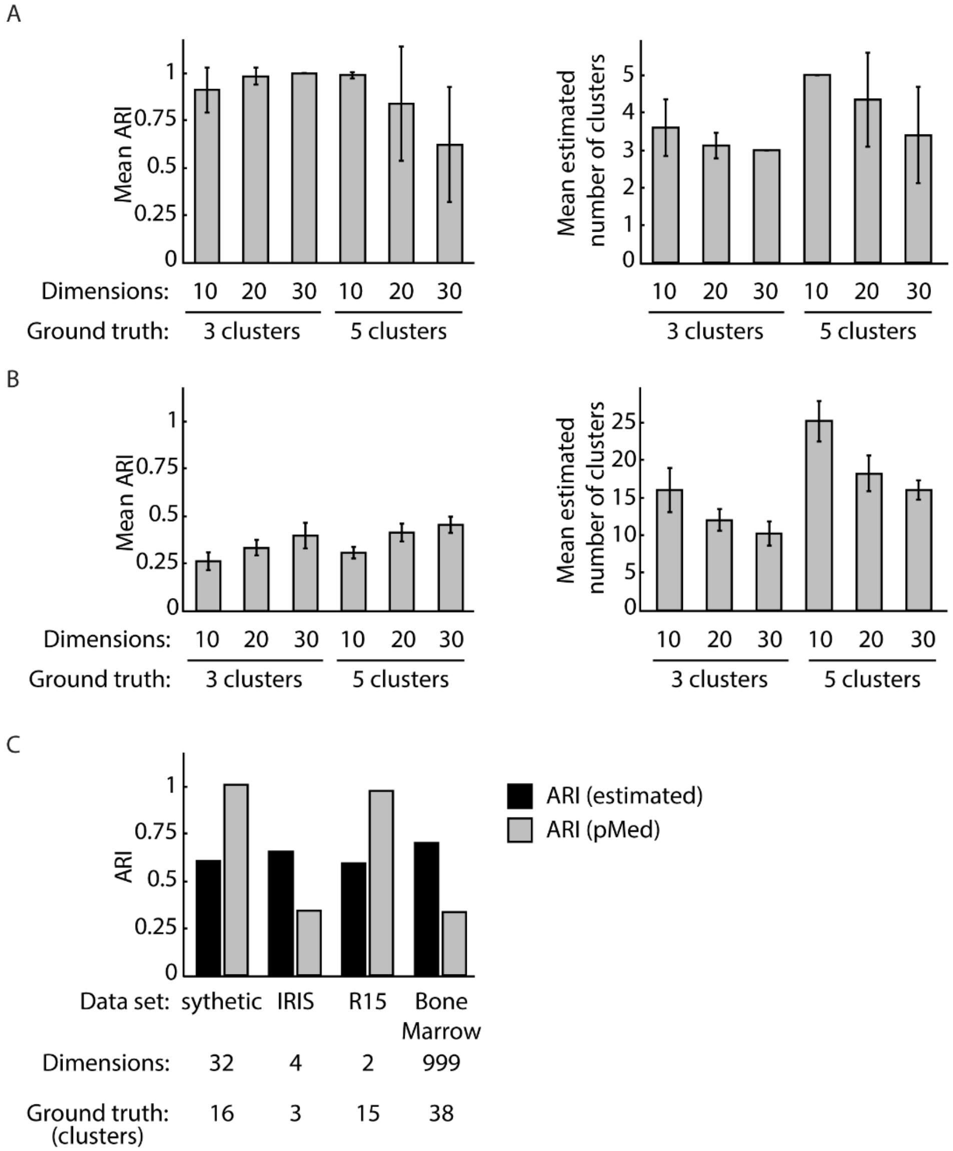

Fig B: Validation for the technique for determination of number of clusters in Phindr3D. In all clustering algorithms, determining a meaningful number of clusters to represent the data appropriately is problematic and generally determined by trial and error. In AP clustering the number of clusters is determined by a variable called the preference value. For AP clustering, Phindr3D automatically estimates the number of clusters from the data by estimating the preference value beyond which the number of clusters increases exponentially (see Fig 5 and methods for additional details). This approach employs an angle based method for knee point detection [1]. Empirical validation of the algorithm for determining a meaningful number of clusters was performed using published and our own gold standard data sets with established ground truths. The graphs show the adjusted RAND index (ARI), a measure for the accuracy of cluster estimation [2]. A value of 0 would indicate a random result and a value of 1 perfect agreement with the ground truth. Generally, for the type of data analyzed here, ARI values greater than 0.5 indicate successful clustering.

A, B) ARI (left panel) and estimated number of clusters (right panel) for randomly generated Gaussian datasets with 10, 20 or 30 dimensions. For each dimension value, 30 different Gaussian datasets with 3 and 5 clusters were randomly generated using different means and variance each time.

A) The number of clusters was estimated using different preference values for affinity propagation (AP) based on our algorithm for automated detection of the optimum preference value. The agreement between the true clustering and the estimated clustering was then expressed as the ARI (left panel) for every dataset. The right panel shows the resulting number of clusters when

using our algorithm for automated detection of the preference value (n=30 ±STD for mean ARI and mean number of estimated clusters).
B) For comparison, we performed AP clustering on the same datasets using the recommended preference value pMedian [3], which is the median of similarity values between the data points, which results in ARI values (left panel) less than 0.5 in most cases. The right panel shows the resulting number of estimated clusters when using pMedian instead of our algorithm for automated detection of the optimum preference value (n=30 ±STD for mean ARI and mean number of estimated clusters).
C) Published datasets with different dimensions were clustered using the automatic clustering approach implemented in Phindr3D (black bars, estimated) or using pMedian (grey bars, pMed). After clustering, the ARI was computed in each case. While automated cluster estimation performed well for the real world data sets (IRIS, bone marrow), using pMedian instead performed better on the well separated synthetic data sets. Synthetic – a synthetic 32 dimensional dataset with 16 Gaussian clusters [4], IRIS – taxonomic iris flower data set with 4 dimensions and 3 clusters [5], R15 – a synthetic two-dimensional data with 15 clusters [6], Bone Marrow – microarray gene expression data of 999 genes (i.e. dimensions) from 38 leukemia patients [7] (i.e. clusters), see Materials and Methods for detail.
In summary, these data demonstrate that the automated clustering approach in Phindr3D yields reliable results in a variety of different use cases.

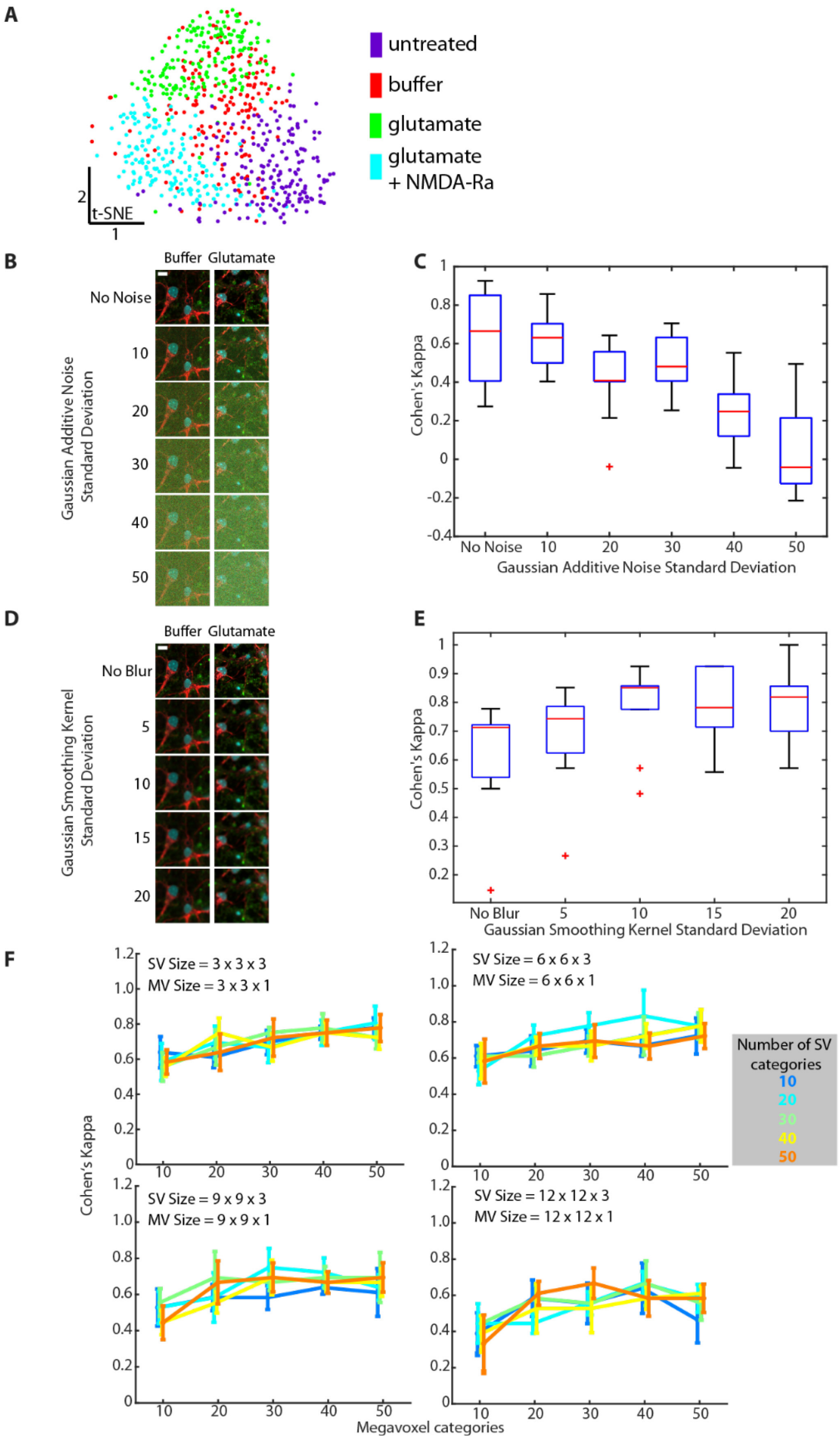

Fig C: Robust performance of data-driven Phindr3D image features.

A) Map of the phenotypic feature space of primary cortical neurons in response to excitotoxic glutamate treatment. Each dot represents one 3D image stack. This control data set was generated to investigate the robustness of the Phindr3D algorithm using an independent complex 3D data set of dense cultured neurons undergoing a well-defined response (excitotoxicity). Plotted is the two-dimensional t-SNE projection of Phindr3D features extracted from 720 three channel 18 stack image data of primary cortical neurons 24 hours after glutamate treatment. As controls, neurons were incubated in BSS0 (buffer) alone or the NMDA receptor antagonists (NMDA-Ra) MK-801 and CNQX were added with glutamate to ameliorate its effects. Prior to imaging, the cells were stained with Mitotracker, annexin V and DRAQ5. The data show a high degree of variability

for the control condition (buffer) with (expected) overlap with the untreated condition and (undesired) overlap in the t-SNE representation into the treatment (glutamate) condition. As expected, the NMDA-Ra-treated condition occupies a different space in the t-SNE than both untreated and glutamate-treated neurons but with some overlap of the space occupied by the untreated and buffer conditions. This is consistent with a scenario where inhibition of glutamate receptors induces a phenotypic change in the cells that is different from the damage induced by glutamate treatment or the phenotypic alterations of the buffer condition. Despite the heterogeneity in the data, the image dataset was useful for measuring the effect of Gaussian noise, 3D Gaussian blur and different values for super-voxel and mega-voxel window size and categories on the performance of Phindr3D features as shown in subsequent figure panels. *Untreated – neurons in medium; buffer – BSS0; glutamate – 25 µM glutamate in BSS0; glutamate + NMDA-Ra - 25 µM glutamate in BSS0 +10 µM MK-801 +10 µM CNQX.*

B, C) Effect of Gaussian random noise on Phindr3D classification performance to distinguish between neurons treated with 25 µM glutamate or incubated in BSS0 (Buffer). Gaussian noise added as random samples with a zero mean and increasing standard deviations resulted in degraded performance, i.e. the level of classification agreement assessed as Cohen's kappa between *No Noise* and *added noise* decreased. Cohen's kappa is a measure of the prediction performance of a classifier (a value of <0 indicates random assignment to a class; a value close to 0.7/0.8 indicates very good agreement). **B)** Sample single plane images of primary cortical neurons stained with Mitotracker (red), annexin V (green) and DRAQ5 (cyan) with increasing Gaussian noise from top to bottom. **C)** Separation of the 25 µM glutamate and BSS0 (buffer) classes assessed as Cohen's kappa over 10-fold cross-validation under the different random noise conditions. With no added noise, Cohen's kappa for these data was ~0.65 while the 25th and 75th percentiles were ~0.4 and ~0.85 reflective of the heterogeneity in the original data set. Adding random Gaussian noise decreased the spread in the data, presumably by reducing the influence of heterogeneous low intensity high frequency (small features) on the automated identification of image features by Phindr3D (see also panels D,E). As the standard deviation of the added noise was increased above 20, feature quality degraded and the separation of the 25 µM glutamate from the BSS0 (buffer) classes diminished. *Box plots show median (red) and 25th and 75th percentiles within the box, 10th and 90th percentiles as whiskers and outliers as individual data points, n=10; scale bar 10 µm.*

D, E) Effect of Gaussian blur on classification performance to distinguish between neurons treated with 25 µM glutamate or incubated in BSS0 (Buffer). Image blurring introduced by smoothing individual images using a Gaussian kernel with dimensions 21, 21 by 5 pixels and different standard deviations. **D)** Sample single plane images of primary cortical neurons stained with Mitotracker (red), annexin V (green) and DRAQ5 (cyan) with increasing added blur from top to bottom. **E)** Cohen's kappa over 10-fold cross-validation under the different blur conditions demonstrated that at standard deviation of 10 image blurring improved classification. This suggests that for this dataset the high frequency information (i.e. small structures) in the images is too heterogeneous to generate useful features (i.e. it is dominated by noise). *Box plots show median (red) and 25th and 75th percentiles within the box, 10th and 90th percentiles as whiskers and outliers as individual data points, n=10; scale bar 10 µm.*

F) Classification performance is insensitive to the numbers of supervoxel (SV) categories but sensitive to the number of megavoxel (MV) categories for varying SV and MV sizes. Classification performance measured as Cohen's Kappa to distinguish between neurons treated with 25 µM glutamate or incubated in BSS0 (buffer). For this dataset there is little difference between SV size 3x3x3 and MV size 3x3x1 and SV 6x6x3 and MV 6x6x1 consistent with the data in panels D-E showing that small structures do not usefully contribute to distinguishing the two treatments. However, increasing the number of MV categories improved performance from Cohen's kappa of 0.6 to 0.8. Each panel shows Cohen's kappa over 10-fold cross-validation for the different parameter conditions. Graphs display n=10 ± SD.

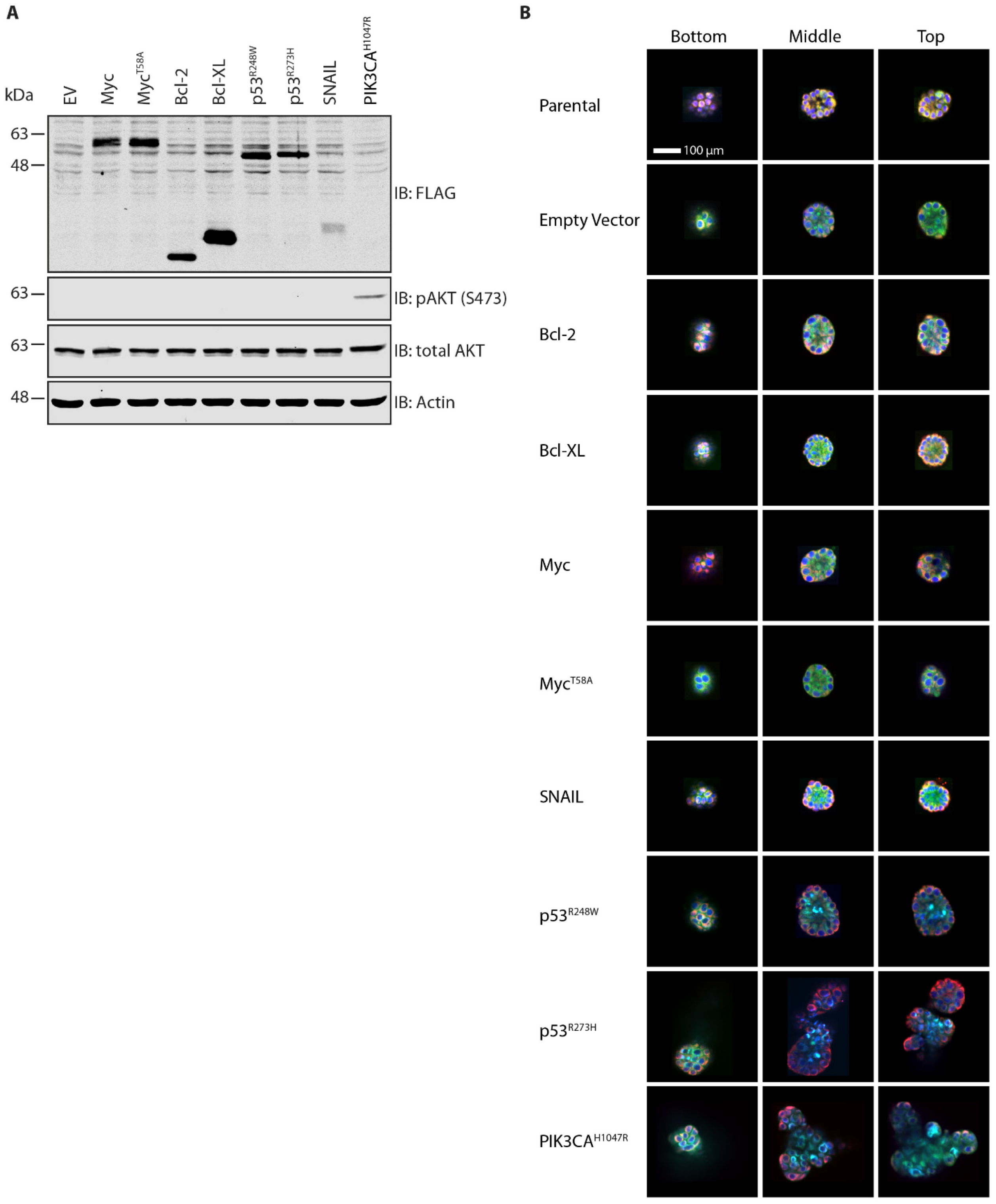

Fig D: Generation of oncogene-expressing MCF10A cell lines and organoids.

A) Western blot of the generated MCF10A cell lines confirming transgene expression. For Myc, Myc^{T58A}, Bcl-2, Bcl-XL, p53^{R248W}, p53^{R273H}, and SNAIL, we confirmed transgene expression using an antibody against the FLAG epitope. For PIK3CA^{H1047R}-ovexpressing MCF10A cells, we confirmed transgene expression using an antibody against the phosphorylated form of the PIK3 substrate AKT (pAKT (S473)). The blot was stripped and probed for pAKT (S473) and as controls actin and total AKT. **B)** Sample images of MCF10A organoids expressing different oncogenes indicated to the left. For each oncogene, the bottom, middle and top slices for a single organoid are shown. Images are from the stack of confocal micrographs pseudocolored as: *Blue – nuclei (DRAQ5), green – lysosomes (Lysotracker), red – mitochondria (Mitotracker). Scale bar: 100 μm.*

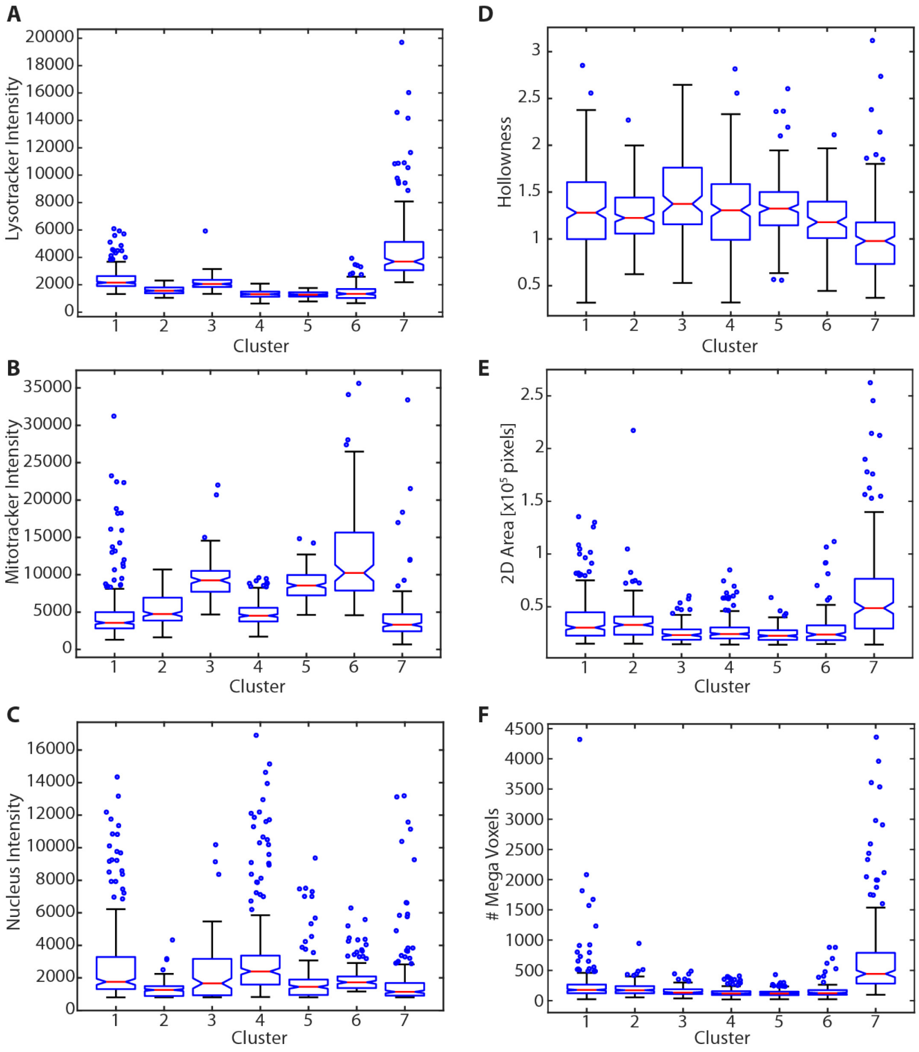

Fig E: 2D intensity- and morphology-based features for each cluster to aid interpretation of Phindr3D feature based clustering.

A-C) Maximum projection based average pixel intensity values for the organoids stained with **A)** Lysotracker, **B)** Mitotracker, and **C)** DRAQ5 (Nucleus Intensity) for each cluster.

D) Hollowness parameter for the different clusters. The hollowness was computed by using the center slice for each organoid and computing the ratio between DRAQ5 intensities of outer ring region (width 20 pixels) and the inner region (that excludes the ring region). A normal acinar organoid is expected to have a high value since the center is lower in intensity. However, there was very little variation in this parameter across the clusters. The reasons for this are not clear.

E) 2D areas computed from 2D binary masks, which corresponds to the conventional way of analyzing this type of data.

F) Number of megavoxels per acinar organoid as a surrogate marker for 3D volume. For this parameter only cluster 7 is significantly different from all other clusters.

Box plots show median (red line) and 25th and 75th percentiles within the box, 10th and 90th percentiles as whiskers and outliers as individual data points. All p-values for group-wise comparison using oneway ANOVA and Tukey-Kramer post-hoc test are shown in Table B in S1 Text.

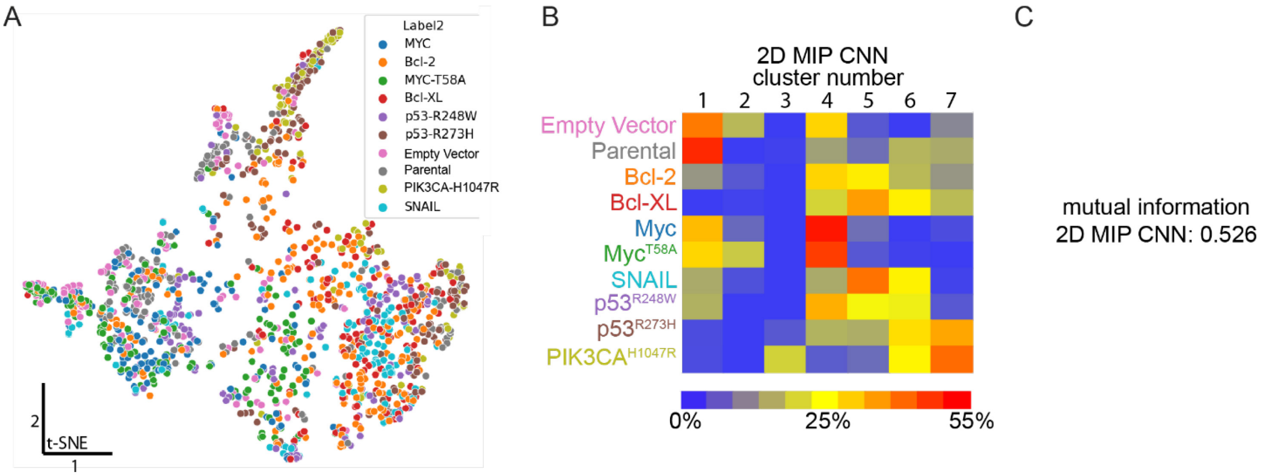

Fig F: Deep learning analysis of oncogene-expressing organoid images

After maximum intensity projection of the image data, a convolutional neural network (CNN) was trained using 500 of the 1330 MCF10A organoid image stacks across the 10 oncogene classes. 30 images per class were chosen for validation.

A) t-SNE mapping of the embeddings of the CCN highlighting the heterogeneity of the resulting phenotypes. The coloring corresponds to the different genotypes.

B) Clustering control and oncogene-expressing organoids using the seven phenotypes identified by Phindr3D (Fig 6C-D) demonstrated that the clustering after deep learning analysis was in good agreement to the clustering after Phindr3D analysis for many (e.g. Empty Vector, Parental, Myc, Myc^{T58A}, $PIK3CA^{H1047R}$), but not all genotypes.

C) The mutual information (MI) of this clustering was calculated with 0.526 and is comparable with the MI generated with Phindr3D (0.516, Fig. 6H).

Table A: Composition of clusters of oncogene-expressing MCF10 organoids.

A) Distribution of the number of oncogene-expressing organoids across the seven clusters.

	Custer 1	Cluster 2	Cluster 3	Cluster 4	Cluster 5	Cluster 6	Cluster 7
**Parental**	62	12	4	14	2	29	26
**Empty Vector**	39	24	6	16	2	0	32
**Bcl-2**	46	29	62	18	39	1	6
**Bcl-XL**	13	31	30	14	25	4	9
**Myc**	28	36	1	57	10	0	4
**Myc^{T58A}**	35	25	0	65	13	0	2
**SNAIL**	12	19	5	15	47	13	2
**p53^{R248W}**	7	24	3	12	24	34	3
**p53^{R273H}**	17	12	12	0	16	25	55
**PIK3CA^{H1047R}**	14	8	1	0	7	20	52

B) Distribution of the percentage of oncogene-expressing organoids across the seven clusters. The fractions were calculated from the number of organoids for each cell line (panel A).

	Custer 1	Cluster 2	Cluster 3	Cluster 4	Cluster 5	Cluster 6	Cluster 7
**Parental**	42	8	3	9	1	19	17
**Empty Vector**	33	20	5	13	2	0	27
**Bcl-2**	23	14	31	9	19	0	3
**Bcl-XL**	10	25	24	11	20	3	7
**Myc**	21	26	1	42	7	0	3
**Myc^{T58A}**	25	18	0	46	9	0	1
**SNAIL**	11	17	4	13	42	12	2
**p53^{R248W}**	7	22	3	11	22	32	3
**p53^{R273H}**	12	9	9	0	12	18	40
**PIK3CA^{H1047R}**	14	8	1	0	7	20	51

Table B: Tables of p-values (Pval) **for Fig E.** Tukey-Kramer post-hoc test performed after oneway ANOVA on the indicated data from Fig E. P-values < 0.05 are highlighted in bold.

		Lysotracker Intensity	Mitotracker Intensity	Nucleus Intensity	Hollowness	2D Area	Num MV
Cluster	Tested against Cluster	Pval	Pval	Pval	Pval	Pval	Pval
1	2	**3.71E-08**	0.364	**3.71E-08**	0.378	0.955	0.303
1	3	0.598	**3.71E-08**	0.277	**0.004**	**2.87E-05**	0.089
1	4	**3.71E-08**	0.999	**9.04E-03**	0.999	**1.36E-05**	**4.73E-04**
1	5	**3.71E-08**	**3.71E-08**	**3.46E-06**	0.999	**4.44E-08**	**5.58E-04**
1	6	**3.71E-08**	**3.71E-08**	**7.85E-03**	**0.025**	**0.013**	0.078
1	7	**3.71E-08**	0.358	**1.43E-05**	**3.71E-08**	**3.71E-08**	**3.71E-08**
2	3	**6.37E-05**	**3.71E-08**	**1.50E-04**	**3.30E-06**	**0.002**	0.976
2	4	0.092	0.593	**3.71E-08**	0.734	**0.003**	0.437
2	5	**0.047**	**3.71E-08**	0.168	0.227	**7.86E-06**	0.406
2	6	0.877	**3.71E-08**	**0.018**	0.806	0.177	0.971
2	7	**3.71E-08**	**1.85E-03**	0.075	**1.44E-07**	**3.71E-08**	**3.71E-08**
3	4	**3.71E-08**	**3.71E-08**	**1.47E-05**	**0.002**	0.997	0.984
3	5	**3.71E-08**	0.458	0.290	**0.035**	0.996	0.975
3	6	**3.38E-06**	**3.71E-08**	0.933	**1.19E-07**	0.873	0.999
3	7	**3.71E-08**	**3.71E-08**	0.439	**3.71E-08**	**3.71E-08**	**3.71E-08**
4	5	0.999	**3.71E-08**	**3.71E-08**	0.978	0.803	0.999
4	6	0.929	**3.71E-08**	**4.82E-08**	0.103	0.983	0.987
4	7	**3.71E-08**	0.301	**3.71E-08**	**3.71E-08**	**3.71E-08**	**3.71E-08**
5	6	0.823	**3.71E-08**	0.946	**0.014**	0.405	0.979
5	7	**3.71E-08**	**3.71E-08**	0.999	**3.71E-08**	**3.71E-08**	**3.71E-08**
6	7	**3.71E-08**	**3.71E-08**	0.987	**0.004**	**3.71E-08**	**3.71E-08**

Supplementary References

1. Zhao Q, Xu M, Fränti P. Knee Point Detection on Bayesian Information Criterion. 2008 20th IEEE International Conference on Tools with Artificial Intelligence. 2008;2:431-8.

2. Vinh NX, Epps J, Bailey J. Information Theoretic Measures for Clusterings Comparison: Variants, Properties, Normalization and Correction for Chance. J Mach Learn Res. 2010;11:2837-54.

3. Frey BJ, Dueck D. Clustering by passing messages between data points. Science. 2007;315(5814):972-6.

4. Franti P, Virmajoki O, Hautamaki V. Fast agglomerative clustering using a k-nearest neighbor graph. IEEE Trans Pattern Anal Mach Intell. 2006;28(11):1875-81.

5. Fisher RA. The use of multiple measurements in taxonomic problems. Ann Eugenic. 1936;7:179-88.

6. Veenman CJ, Reinders MJT, Backer E. A maximum variance cluster algorithm. Ieee T Pattern Anal. 2002;24(9):1273-80.

7. Monti S, Tamayo P, Mesirov J, Golub T. Consensus clustering: A resampling-based method for class discovery and visualization of gene expression microarray data. Machine Learning. 2003;52(1-2):91-118.
