# Supplementary material for: Rapid 3D phenotypic analysis of neurons and organoids using data-driven cell segmentation-free machine learning
Source: PLoS Comput Biol. 2021 Feb 22;17(2):e1008630. doi: 10.1371/journal.pcbi.1008630 (PMC7932518; doi:10.1371/journal.pcbi.1008630)
Supplement: Attachment — Submitted filename: Phindr3D_PlosCompBiol_Reply to Reviewers_v4_final.docx [file pcbi.1008630.s002.docx]

**REPLY TO REVIEWER COMMENTS**

**Reviewer #1:**

The paper presents a segmentation-free voxel classification approach to phenotypic profiling in 3D microscopy data. While the general idea of recursive supervoxel clustering for image classification is not new, and actually constitutes a standard approach in computer vision (multi-resolution pyramids), the application here seems significant and the work is of high practical value for users, in particular because a software is made available.

We thank the reviewer for this encouraging comment. We have added a reference to spatial pyramids in computer vision (new reference 11).

Suggestions for improvement:

1) The basic motivation should be toned down a bit. It is not true that phenotypic profiling of “three-dimensional microscopy datasets has been impractical”. It has actually been done before. It may not be easy, though.

We have revised the wording accordingly. In our introduction, we had already highlighted some of the current applications and discussed that “Analyses of three-dimensional (3D) organoid cultures or complex cellular phenotypes, such as in dense neuronal cultures, are not easy with conventional methods.”

2) It may be good to state the amount of training data needed for “shallow learning” right at the beginning of the results section in order to justify the use of the term “shallow learning”, which has a well-defined meaning in the machine-learning community.

The amount of training data can be adjusted by the user. By default, Phindr3D uses 10 images for training. We have added that information in the results section as suggested (line 96). With this amount of training data, Phindr3D is able to generate results with comparable accuracy to conventional cell segmentation based methods (Figure 2). Furthermore, Phindr3D generates results with mutual information comparable to a deep learning convolutional neural network that required 500 images for training (please see the new figure Fig F of S1 Text and our reply to reviewer 2 below for further details).

3) In line 107, I would appreciate some information on how the binning is done. Based on which pixel property is the data binned? How many bins?

Binning is based on normalized pixel intensities. For every pixel, a vector of normalized pixel intensities is generated, and K-means clustering is applied. The bins are set to 20 by default but can be adjusted by the user. We have added this to the manuscript accordingly (now line 114).

4) The observation that adding Gaussian noise to the data first improves performance is surprising. Could the authors elaborate on why this is? Is the classifier partially picking up noise signatures? Would performance on perfectly noise-free (synthetic) images be worse?

We assume that this comment is in respect to panel C in Fig C of S1 Text. In general, the features and thus the classifier are obviously affected by increased noise. However, even though the distribution of Cohen’s Kappa is a little bit smaller with Gaussian Additive Noise at a standard deviation of 10, the mean value (red bar) is almost the same as without addition of noise. We interpret this such that there is no measurable difference between these two groups. Further addition of noise leads to decreased Cohen’s Kappa and thereby indicates poorer performance of the classifier.

5) I cannot follow the claim made in the Discussion section that the method used here to determine the number of clusters is novel. Nor do I believe that it is optimal (with respect to which loss function?). It seems that the authors are using the classic L-curve method for determining the number of clusters. This method is known to be a heuristic without any optimality guarantees on the test data, and it is well known. This claim should be removed, or it should be explained why and how the methods differ.

We did not mean to imply that the method to determine the number of clusters would result in a mathematically “optimal” number of clusters but rather we intended to imply that this method would result in a number of biologically meaningful (i.e. “biologically optimal”) clusters. In that regard, it was our aim to avoid that a researcher would arbitrarily have to choose a number of clusters. We have revised the wording throughout to more appropriately reflect the intended meaning.

In our implementation of the method to determine the number of clusters, we use the angle-based method for knee point detection as described by Zhao et al. (*Zhao Q, Xu M, Fränti P. Knee Point Detection on Bayesian Information Criterion. 2008 20th IEEE International Conference on Tools with Artificial Intelligence*), which we had referenced in the methods section of our original manuscript. We thank the reviewer for highlighting the need for a reference in the main text which we added in the results section (line 210) as well as in the figure legend for Fig B of S1 Text. Furthermore, we have removed the word “novel” in the sections talking about the implementation and validation of this algorithm.

6) The discussion section would benefit from being a bit more self-critical and also highlight the limitations of the method, or cases where such analysis would fail altogether. Clearly, the size of the supervoxels and megavoxels sets a resolution limit. I can also not imagine this method to be useful to classify phenotypes that mainly differ in morphology or shape. Nor would the method be able to distinguish different textures with the same intensity frequencies. It would be good if the authors could provide some guidelines for when someone should consider their method, to reduce the risk of user frustration.

Our goal was to establish an image processing approach that would capture phenotypic differences that were specifically not dependent on shape / morphology. Indeed, we show that merely using shape descriptors results in poor clustering of the resultant data compared to using Phindr3D, at least when they are applied in 2D. However, some morphology information can be obtained by Phindr3D indirectly, e.g. by using the number of megavoxels as a morphology descriptor. Indeed, Phindr3D will be sensitive to the spacing between the z-slices of the 3D image stacks. For Phindr3D to perform best, z-slices should be spaced at Nyquist resolution. Thus, spacing larger than that will lead to loss of resolution and in turn affect the output.

At the beginning of the discussion section, we now provide some guidance on experimental approaches where we think Phindr3D will be of utility. We had already described some of these details in the methods and results sections and we have added a short sentence pointing the reader to these sections.

7) From a technical point of view, I would not call the method presented here “segmentation-free”. In fact, one of the first steps in the proposed processing pipeline is to apply adaptive thresholding to the images in order to distinguish foreground pixels from background pixels. This *is* segmentation. It may not be instance segmentation, but it is the classic definition of image segmentation, and thresholding is known to be the simplest possible segmentation algorithm. In this sense, the method presented is not segmentation-free, and one may even ask the question whether it could be improved by using better (i.e., information-optimal) initial segmentation methods. This should be discussed and/or the language corrected.

We have changed the language to “cell segmentation independent” to reflect that we are thinking of segmentation in the context of a biological system. While it may be that for the analysis of organoids where the initial image segmentation removes the image areas that do not contain objects the border region constitutes a small part of the object analyzed and is therefore unlikely to be altered by using better initial segmentation methods. In images of dense cortical neurons it is now possible to identify neuronal morphology in (electron microscopy) images using deep learning (Schubert et al., Learning cellular morphology with neural networks, Nat Commun 10, 2736 (2019)). However, this remains a non-trivial exercise that is not practical for most biological researchers that do not have access to the expert knowledge required for performing segmentation of complex cellular structures in 3D. Importantly the limited resolution, particularly in Z, in fluorescence micrographs means that segmentation is still problematic.

In essence, what I think is done here, is weakly-supervised shallow-learning of intensity histograms in a 3-level multi-resolution pyramid over pre-segmented images. Nothing of this is new by itself, but the presented pipeline is practically useful.

We thank the reviewer for agreeing with us on the practical utility of Phindr3D for three-dimensional phenotypic high content profiling. To the best of our knowledge and literature review, using pixel-based information for phenotypic analysis of a large number of images in 3D has not yet been performed. While the Phindr3D approach may not be appropriate for every conceivable image profiling problem, our intent was to provide a facile tool for first line analysis of complex fluorescence microscopy data. In Figure 2 of the manuscript, we demonstrate that the Phindr3D approach of cell segmentation independent image profiling in 3D using pixel-based feature learning yields comparable results to conventional cell segmentation-based analysis using preconceived features. As referred to in our replies above, we have added information / discussion to the manuscript text to reflect the valid arguments raised by this reviewer. Further, we have added Lazebnik et al. 2006 (new Ref. 11) as a reference to spatial pyramids.

**Reviewer #2:**

The main contribution of this manuscript is a novel, super-pixel-based method that circumvents the need to explicitly segment structures and pre-define quantitative features when analyzing 3D micrographs of organoids.

The methodology is described well but the use of fixed pixel sizes to create super-voxels and mega-voxels will make the method dependent both on the magnification of the image and the size of the organoids. A similar problem exists for the method of focus stacking.

Thank you. It is true for Phindr3D as well as many other image analysis systems where fixed pixel sizes are used and is a limitation of our approach. We have pointed this out to readers on pages 15 and 21.

The authors argue that their method is superior to deep learning methods (DLMs) because of lower computational resource needs. However, this argument is questionable because DLMs are becoming state of the art and the cost of their computational resources are well within the reach of scientific researchers and in some cases are free.

Moreover, DLMs might significantly outperform the proposed method for the same tasks, in which case a DLM would be worth it even if it costs more. Therefore, the authors need to compare their method with a state of the art DLM. This is one of the two major shortcomings of the study.

While the reviewer is of course correct in arguing that DLMs have found their application in many aspects of computational microscopy image analysis, we were unable to identify a proper DLM for comprehensive 3D high content analysis. We have added references and information in the introduction and in the discussion.

Therefore, to compare performance of Phindr3D to a DLM, we used a convolutional neural network (CNN) for classifying maximum intensity projected images of the mammary acinar gland organoid 3D image dataset. Each image was either cropped or padded to have a final image size of 256x256 pixels throughout. We trained the CNN using 10 classes based on the 10 different genotypes of the organoids and a total of 500 images. We used the test data to plot a confusion matrix with an accuracy of ~10%. Next, we extracted the embeddings and used these data to cluster the data into 7 different clusters as with Phindr3D. We found that the clustering after DL analysis was in good agreement to the clustering after Phindr3D analysis for many (e.g. Empty Vector, Parental, Myc, Myc^T58A^, PIK3CA^H1074R^), but not all genotypes. The mutual information of this clustering was calculated with 0.526 and is essentially identical with the MI generated with Phindr3D (Figure 6H).

We here provide the confusion matrix for the scrutiny of the reviewer. We show a t-SNE map of the embeddings, a heatmap of the clustering results, and the mutual information for the clustering in the new supplemental figure Fig F of S1 Text.

Confusion matrix of the classification results (columns) after CNN analysis of the mammary acinar gland organoid image data. Numbers indicate individual images of a specific organoid genotype (rows).

While we do not wish to argue that Phindr3D would outperform DLMs, it is our argument that Phindr3D provides facile access to advanced methods for high content profiling that generate high quality data without the need to employ DLM. In addition, Phindr3D is much less demanding on the amount of training data. Furthermore, our experience in the course of this revision shows that a high degree of expert knowledge is still required to run and optimize a deep learning neural network. We agree with the reviewer that depending on the circumstance no cost may be too high to access the most advanced DLM. However, for many biological research laboratories, which we anticipate will find good utility of Phindr3D, the opportunity costs for using DLMs may be too high. Certainly, it would not be used as a rapid data exploration tool in screening as we have proposed as one of the main uses for our approach.

There needs to be some discussion about the choice of principal component analysis and Sammon mapping versus tSNE and UMAPP for projecting the data.

In Phindr3D we elected to use principle component analysis (PCA), Sammon mapping, and tSNE as tools for displaying the multidimensional feature space. While there is no metric to determine the ideal method to project the data, we allow the user to choose the method that most appropriately highlights the structure of the respective data. We have added a short section highlighting the most basic differences between the three methods upon first mention (line 123).

The claim based off figures 5D, 5E and 5F that Phindr3D is better than the others is not apparent. The improvement from using Phindr3D needs to be quantitatively compared to the other methods and shown to be statistically significant.

Please note, that we split Figure 3 into two figures (now Figure 3 and Figure 4) to better comply with the editorial standards for figure preparation of Plos Computational Biology. Therefore, previous Figure 5 is now Figure 6.

Respectfully, we are unsure why the reviewer argues that it is not apparent that Phindr3D (Fig 6D) outperforms clustering based on 2D morphological features (Fig 6E) or 3D texture features (Fig 6F). It has been discussed that there is no acknowledged metric of choice to compare different clusterings (*Wu et al., Proceedings of the 15th ACM SIGKDD international conference on Knowledge discovery and data mining (KDD '09), 2009; Romano et al., Proceedings of the 31st International Conference on Machine Learning, 2014*). In our paper, we compute mutual information, a metric derived from information theory, to determine the probability whether clustering accurately reflects real differences in the data. According to this metric Phindr3D outperforms the other methods mentioned in Fig 6. Moreover, as discussed in the manuscript the correspondence of the Phindr 3D clustering with the known biology of the system is much better than with the other methods.

The biological results are quite impressive and interesting, but they only confirm what was anticipated. The value of the proposed methodology can only be proven by demonstrating that the method produces new biological insights that could not have been obtained from using other, existing methods that are approximately equivalent in terms of cost (human effort, computer hardware costs, reagent costs etc). This is the second major shortcoming of the manuscript.

We agree with the reviewer that the biological results of the studies that Phindr3D enabled us to do are very interesting. However, we respectfully disagree with the notion that the results were anticipated. Indeed, the results provide novel and unanticipated biological insight.

First, when treating primary neurons with different inhibitors of antiapoptotic Bcl-2 family inhibitors (Fig. 3, Fig. 4), we hypothesized that functional complementarity between these antiapoptotic proteins would result in similar cell death phenotypes upon their inhibition. We therefore anticipated that the resulting cell death phenotypes (in all cases the drugs should induce apoptosis, a morphologically defined cell death program) would all follow the same vector on a concentration-response gradient. However, while we found a clear concentration-dependent increase of apoptotic cell death, as expected, we also discovered distinct and previously unknown apoptotic phenotypes elicited by inhibition of the different anti-apoptotic proteins in primary. The latter is indicated by occupancy of different areas in the PCA map of the different experimental groups (Fig. 4A). We have revised the text to make the new insights clearer to readers.

Second, using mammary gland acinar organoids we demonstrate a previously unknown large degree of phenotypic heterogeneity in the organoids generated by the cell line that increased upon oncogene expression in these organoids (Fig. 6), which is indicative of various phenotypes for each oncogene genotype. Moreover, our analysis is the first attempt to quantify this heterogeneity. The current approaches being used to analyze heterogeneity in these types of cultures include single cell RNA sequence analysis, multicolor flow cytometry and spatial trascriptomics. Single cell RNAseq is hundreds of times more expensive and requires months of analyses by bioinformatics experts. Multicolor flow cytometry is expensive and convolutes cell responses with anoikis due to detachment from substrate and cannot be used with dense neuronal cultures. Only spatial transcriptomics preserves spatial relationships in dense cultures but cannot at present be used productively with 3D cultures, is expensive and limited primarily to cell type identification. For the organoid cultures here it would yield only ‘epithelia’ as the analytic result.

To the best of our knowledge, there are no other existing methods that would enable phenotypic high content profiling in 3D of complex structures such as dense neuronal cultures and organoids. While in principle other assays such as biochemical approaches would allow performing concentration response analyses, 3D high content microscopy enables this analysis without the need of using a large amount of scarce cell types. As we demonstrate in Figure 6 of the manuscript, using preconceived morphology features in 2D or preconceived texture features in 3D yields results inferior to the results produced using Phindr3D.

**Reviewer #3:**

The manuscript of Mergenthaler et al introduces a software tool termed Phindr3D for automatic extraction of features from 3D microscopy image stacks. The software targets the problem of whole-image (in this case, whole-3D-image) classification and clustering, with classification and clustering methods based on the extracted features also provided in the software. The algorithm is applied to several biological problems where it produces results consistent with the prior knowledge.

We would like to thank the reviewer for the positive remarks on our manuscript. However, as elaborated in our reply to the final comment of reviewer 2, we would like to point out that we also discovered unanticipated novel cell death phenotypes in complex cultures of primary neurons after chemical biology inhibition of anti-apoptotic Bcl-2 family proteins. Furthermore, we discovered a previously unknown degree of phenotypic heterogeneity in human mammary gland acinar organoids upon oncogene expression, which is a model of breast cancer.

The work has a lot of positive sides. All the data will be available at IDR, the software is provided (but I couldn't find a way to run it on Mac or Linux without a Matlab license which I believe most potential Phindr3D users are not going to possess), application is demonstrated on several very different phenotyping problems, the method itself is fast and scales well.

We thank the reviewer for this encouraging comment. We are working on making Phindr3D more usable across operating systems. However, at present a Matlab license with Image Processing and Statistics and Machine Learning toolboxes are required to run Phindr3D on Mac and Linux operating systems.

Furthermore, in addition to providing the entire data sets generated in this study through IDR, we will provide the test data sets that were available for the reviewers through Zenodo, an open science platform supported by the CERN and the European Commission. All uploads are currently in progress and we will provide DOIs as soon as we have them.

My main problem with the manuscript is that it presents itself as a novel computer vision method. Image classification based on spatial pyramids is a very well studied area. The seminal work of Lazebnik et al from CVPR2006 (Beyond Bag of Features: Spatial Pyramid Matching for Recognizing Natural Scene Categories) has been cited around 10,000 times, many of these citations improving the original method which is already quite close to what the authors are proposing.

We thank the reviewer for pointing out this reference. We have added it in the introduction section of the manuscript (new Ref. 11).

This whole sub-field, along with other explicit image feature methods, has largely been replaced by CNN-based classification, which, at least on natural images, has been shown too be vastly superior. The authors choose not pursue the deep learning route, as they want to run their software on regular desktop PCs without GPUs (this is how I understand their reasoning), but all this existing work needs to be acknowledged thoroughly. One small difference is that the authors operate in 3D rather than 2D, but since the binning they perform is regular this extension is straight-forward.

Phindr3D aims at quick processing of 3D microscopy data for high content analysis applications performed by biologists without the need to modify image datasets or expert knowledge in deep learning. We found several deep learning 3D methods for instance segmentation. However, feature extraction of the 3D datasets was still performed using pre-engineered features. Furthermore, we were unable to identify a framework for comprehensive 3D high content analysis using deep learning. We therefore compared Phindr3D to a convolutional neural network for analyzing maximum intensity projected images of the mammary acinar gland organoid 3D image dataset. For details and discussion, we kindly refer this reviewer to our reply to reviewer 2 and to the changes made in the manuscript (Fig F in S1 Text and corresponding sections in the Results and Discussion sections). In the course of performing this deep learning based analysis for this revision, we found that employing deep learning for the specific task of 3d high content analysis is still a very challenging undertaking without expert knowledge. Moreover, it did not improve the results significantly.

Finally, we have added a brief segment in the introduction and discussion on applications of deep learning in biological microscopy image analysis.

I am not a biologist and can not comment on the biological applications, I hope other reviewers can address these points.

________________________________

Have all data underlying the figures and results presented in the manuscript been provided?

Large-scale datasets should be made available via a public repository as described in the PLOS Computational Biology data availability policy, and numerical data that underlies graphs or summary statistics should be provided in spreadsheet form as supporting information.

Reviewer #1: Yes

Reviewer #2: No: The manuscript clearly states, "All 3D microscopy data sets generated in this study WILL BE deposited ...."

We are currently in the process of transferring the microscopy data sets to IDR and Zenodo. We will provide DOIs for all datasets as soon as we receive them from the repositories.

Reviewer #3: Yes

________________________________
